# Perceived clinical challenges when treating patients from another culture: a study among doctors training in psychiatry in Norway

**DOI:** 10.1192/j.eurpsy.2022.524

**Published:** 2022-09-01

**Authors:** R. Tyssen, M. Sandbu, S. Thapa, K. Rø, C. Jávo, V. Preljevic

**Affiliations:** 1 Institute of Basic Medical Sciences, Faculty of Medicine, University of Oslo, Department Of Behavioural Medicine, Oslo, Norway; 2 Oslo University Hospital, Regional Section For Eating Disorders, Oslo, Norway; 3 University of Oslo, Institute Of Clinical Medicine, Division Of Mental Health And Addiction, Oslo, Norway; 4 Institute for studies of the medical profession, Research Unit, Oslo, Norway; 5 Finnmark Hospital Trust, Sami National Competence Center For Mental Health And Addiction (sanks), Karasjok, Norway; 6 Health South-East, Institute Of Psychotherapy, Oslo, Norway

**Keywords:** Postgraduate training, Assessment in psychiatry, Transcultural psychiatry, Physician role

## Abstract

**Introduction:**

There is increased migration of patients and physicians worldwide. In Norway, psychiatry is the medical discipline with highest proportion of foreign doctors (24%). We need empirical studies on transcultural clinical challenges among doctors training in psychiatry.

**Objectives:**

What perceived clinical challenges do foreign and native Norwegian young doctors meet when they treat patients from another culture, and what independent factors are associated with such challenges?

**Methods:**

We developed a new 6-item instrument (alpha=0.80), Clinical Transcultural Challenges (CTC), with items about assessing psychosis, risk of suicide, violence etc. The doctors were recruited at mandatory training courses, and they filled in questionnaires about individual factors (age, gender, foreign/native) and work-related factors (training stage, frequency of transcultural meetings, number of working hours, work stress). Associations with CTC were analyzed by linear multiple regression.

**Results:**

The response rate was 93% (216/233), of whom 83% were native and 17% were foreign doctors, 68% were women. Native doctors reported higher levels of CTC than did foreign doctors, 28.8 (6.2) vs 23.8 (7.2), p<0.001, d=0.73. Both native and foreign doctors rated “assessing psychosis” and “lack of helping tools” as most demanding. Independent factors associated with CTC were being a native doctor, Beta 3.9, p<0.01, and high levels of work-home stress, Beta 0.29, p<0.05.

**Conclusions:**

Native doctors training in psychiatry report higher levels of transcultural clinical challenges than foreign doctors do. Both groups of doctors may need more training in transcultural assessment of psychotic disorders. They also report needs for more helping tools, and we should explore this further.

**Disclosure:**

No significant relationships.

